# 24-Hour movement behaviours research during the COVID-19 pandemic: a systematic scoping review

**DOI:** 10.1186/s12889-023-17136-y

**Published:** 2023-11-07

**Authors:** Danqing Zhang, Sitong Chen, José Francisco López-Gil, Jintao Hong, Fei Wang, Yang Liu

**Affiliations:** 1 School of Physical Education, Shanghai University of Sport, Shanghai, 200438 China; 2 Institute for Health and Sport, Victoria University, Melbourne, VIC 8001 Australia; 3 One Health Research Group, Universidad de Las Américas, Quito, 170124 Ecuador; 4 Shanghai Research Institute of Sports Science (Shanghai Anti-doping Agency), Shanghai, 200030 China; 5 Kun Shan Lu Jia Senior High School, Jiangsu, 215331 China; 6 Shanghai Research Centre for Physical Fitness and Health of Children and Adolescents, Shanghai University of Sport, Shanghai, 200438 China

**Keywords:** 24-hour movement guidelines, The COVID-19, Moderate to vigorous physical activity, Sedentary behaviour, Sleep, Evidence synthesis

## Abstract

**Objectives:**

Many studies examining 24-hour movement behaviours based on the 24-Hour Movement Guidelines (24HMG) have been published during the COVID-19 pandemic. However, no comprehensive reviews summarized and synthesized the evidence concerning studies using 24HMG. The aim of this scoping review was to synthesize the evidence from the 24HMG studies published during the pandemic.

**Methods:**

Three electronic databases (Web of Science, PubMed, EBSCO) were utilized to conduct a literature search. The search procedure adhered to the guidelines set by the Preferred Reporting Items for Systematic Reviews and Meta-Analyses (PRISMA). Initially, a total of 1339 research articles published in peer-reviewed journals were screened. After eliminating 461 duplicates, 878 articles remained. The titles and/or abstracts of these articles were then cross-checked, and 25 articles were included. Subsequently, two authors independently assessed full-text of articles based on the pre-defined inclusion and exclusion criteria, resulting in the final selection of 16 articles that met the inclusion criteria. Study characteristics (e.g., study population, study design, measurement) were extracted and then summarized. According to the Viable Integrative Research in Time-use Research (VIRTUE) epidemiology, the included studies were further classified into different but interrelated study domains (e.g., composition, determinants, health outcomes).

**Results:**

The majority of included articles focused on children and adolescents as study population. This study primarily demonstrated that a low prevalence of meeting the 24HMG among children and adolescents during the COVID-19 pandemic. There has been a decline in the percentage of individuals meeting the 24HMG compared to the pre-COVID-19 period. The majority of included studies focused on sociodemographic factors when examining the correlates of meeting the 24HMG, while a few studies assessed factors of other domains, such as social, cultural, and environmental aspects.

**Conclusion:**

The COVID-19 pandemic had an impact on healthy 24-hour movement behaviours in children and adolescents. In conjunction with the studies conducted during the COVID-19 pandemic, more studies were encouraged to explore the correlates of meeting the 24HMG and the associated health benefits in wider ranges of populations.

**Supplementary Information:**

The online version contains supplementary material available at 10.1186/s12889-023-17136-y.

## Introduction

The coronavirus disease 2019 (COVID-19) causes a high morbidity and mortality rate and severely affects the world [1]. The World Health Organization (WHO) announced COVID-19 as a pandemic in March 2020. To prevent and limit the possible spread of COVID-19, the governments of some countries issued a series of restrictive measures [2–5], including the suspension of school, work organized sports activities and meetings (though allowing for outdoor activities), and implementing national quarantine, restricting the movement of the entire population [6, 7]. The COVID-19 pandemic has particularly affected people’s lives and health behaviour [3], including home isolation and restrictions on activity accessibility, resulting in significant alterations in daily activities [8, 9]. In addition, the COVID-19 pandemic also increased kinds of risk disease [10, 11]. These circumstances were similar to those of prior disasters [12]. One of the possible reasons for this was the change in lifestyle behaviour after the disaster [2]. Confronted with unparalleled challenges and disruptions to their daily lives, individuals often adapt their routines, habits, and activities. These adaptations can manifest in diverse aspects of lifestyle behavior, including decreased physical activity (PA), modified dietary patterns, and disrupted sleep schedules. Consequently, such modifications in lifestyle behavior can exert a profound and enduring influence on individuals’ overall health and well-being. Additional research is warranted to acquire a comprehensive understanding of the precise changes in lifestyle behavior triggered by the COVID-19 pandemic.

Not surprisingly, it has been well documented that the COVID-19 pandemic has significant impacts on individuals’  PA [14–16], sedentary behaviour (SB) [17–19] and sleep [20–22] (these three behaviours were collectively called 24-hour movement behaviours). During the COVID-19 pandemic, people’s PA levels have shown massive declines [14–16], while SB has shown substantial increases [17–19], primarily owing to social-distancing and lockdown measures [19]. In terms of sleep, studies have shown a longer duration of sleep time and daytime sleepiness [20–22] and adverse changes to sleep patterns and bedtime routines during the home confinement period [20]. These negative changes influence individuals’ health and wellbeing [3].

Given that PA, SB and sleep are co-dependent health behaviours and their combined health effects should be given more research attention rather than focusing on either of them, it is recommended that researchers integrate PA, SB and sleep for efficient health promotion [23, 24]. A well-developed paradigm for 24-hour movement behaviours research was to adopt the 24-hour movement guidelines (24HMG). Researchers have developed and launched the Canadian 24HMG for populations across the life course [24–26]. The guidelines mainly have quantifiable recommendations on PA, SB and sleep, supported by robust scientific evidence. Based on this, an increasing number of studies have begun using 24HMG to study PA, SB and sleep in combination [27–29], as it can help provide an integrative perspective to study movement behaviours at the population level.

Some studies have been conducted using the 24HMG before the COVID-19 pandemic, which examined the prevalence of meeting the 24HMG [30–35] and the secular trends [28, 31, 36–39], correlates of meeting the 24HMG [30, 32, 33, 35, 40], and the associations between meeting the 24HMG and health outcomes [28, 41–44]. According to the Framework for Viable Integrative Research in Time-Use (VIRTUE) Epidemiology, those studies can be categorized into some research areas, time-use compositions, determinants and health outcomes [45].

Given the importance of integrating PA, SB and sleep, a number of studies have investigated the prevalence of meeting the 24HMG during COVID-19 [7, 46–48]. Furthermore, some of the studies repeatedly measured the prevalence of meeting the 24HMG prior to and during COVID-19, enabling researchers to examine the trends of 24HMG adherence. Jáuregui et al. found that the prevalence of meeting the 24HMG was significantly lower than that before COVID-19 [49]. Another study by Angel et al. also had similar research findings, indicated that the percentage of participants meeting the 24HMG has decreased from 3.3 to 0.2% [50]. Despite these studies, there was no synthesized study to review the changes in the prevalence of meeting the 24HMG before and during the pandemic. Such studies were needed not only because of 24-hour movement behaviours associated health outcomes but also to assist with the development of public health interventions when confronting similar public health events.

In addition to the changes in the prevalence of meeting the 24HMG, little was known about which factors (categorization of factors based on VIRTUE framework) were associated with the integrated 24-hour movement behaviours during the COVID-19 pandemic. Therefore, the main aim of this review was to synthesize the evidence concerning research using the 24HMG during the COVID-19 pandemic.

## Methods

This study aimed to conduct a systematic scoping review to summarize the evidence concerning the 24HMG research conducted during the COVID-19 pandemic.

### Data source and search strategy

To ensure a nonbiased and complete review, we searched the following electronic databases from 1 to 2020 to 30 November 2022: Web of Science, EBSCO, and PubMed. Several keywords were employed for the literature search in each database: “24-h*”, “24 hour”, “24-hour”, “Movement Behavio*”, “Sleep*”, “Screen”, “Physical Activity”, “Guideline*”, “recommendation*”, “COVID-19” “Coronavirus Disease”, “Coronavirus”, “SARS-CoV-2” and “nCoV”. In the Web of Science, EBSCO, and PubMed, we divided all search terms into three categories: (24-h* OR 24 h OR 24-hour OR Movement Behavio* OR Sleep* OR Screen OR Physical Activity) AND (Guideline* OR recommendation*) AND (COVID-19 OR Coronavirus Disease OR Coronavirus OR SARS-CoV-2 OR nCoV). Due to the differences in databases, field tags of “Title”, “Abstract” and “Title/Abstract” were used in combination during document retrieval (Supplementary material). To obtain the final number of studies included in this review, all of the retrieved articles were independently screened and assessed by two authors. If there were any differences regarding inclusion, a third author was invited to join the discussion and make a decision.

### Eligibility criteria

The inclusion criteria for screening articles were as follows: (1) documents contained search terms and were published from 1 to 2020 to 30 November 2022; (2) study sample related to human beings, and they were acceptable if the subjects were in poor physical condition (disability or disorder); (3) the results reported combined 24-hour movement behaviours using the guidelines (PA or SB and sleep) focused on people who had COVID-19 at the time of the study; and (4) articles written in English.

The exclusion criteria were as follows: (1) studies that met the inclusion criteria but had duplicates between databases; (2) case studies, master’s/doctoral dissertations, conference papers and abstracts, reviews, brief reports and letters, protocol, commentary, and qualitative study; and (3) studies that did not report the percentage adherence to the 24HMG during the COVID-19 pandemic.

### Data extraction and data items

The following information was extracted and summarized from the final included studies by two authors: (1) basic information of study (authors, published year, published journal, author countries/organization); (2) sample characteristics of study (sociodemographic, subjects, age group and sex of subjects, simple size and population, countries/country of study conducted); (3) study design and method (study design: cross-sectional study/longitudinal study, survey method: single/mixed method); (4) measurements (self-report, interview, device-based measurement, proxy report); (5) categorizations of research areas in line with the VIRTUE framework (including three types of content in this study: outcomes, correlates/determinants, time-use composition of 24-hour movement behaviours with COVID-19); (6) findings (prevalence of meeting % the 24HMG during COVID-19 and changes % of meeting the 24HMG with COVID-19). EXCEL 2019 was used to categorize these variables.

### Coding of studies and summary

The code “D-^*^” indicates studies that reported a significant decrease (%) in meeting the 24HMG between before and during the COVID-19 pandemic period. The code “D-” indicates studies that reported a nonsignificant decrease (%) or reported decreased outcomes (%) but did not report the *p* value. The code “I^+^” indicates studies that reported a nonsignificant increase (%). The code “NC” indicates studies that reported no change (%) in meeting the 24HMG between before, and during the COVID-19 pandemic period. The “Summary” contains a code to summarize the state of studies for meeting the 24HMG. If the study contained fewer than three outcomes, the trend could not be summarized.

## Results

### Data selection

**Fig. 1:**
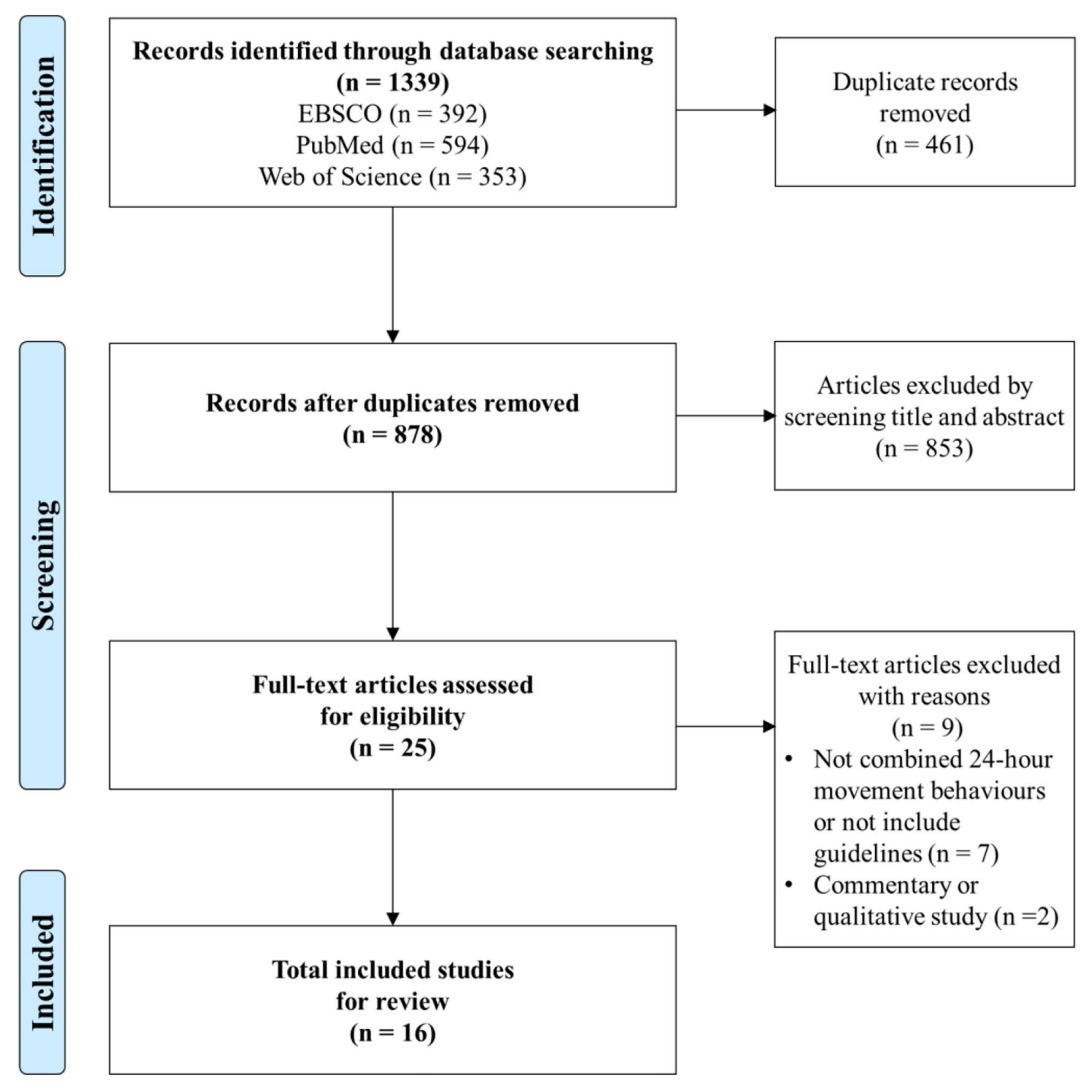
PRISMA flowchart for study selection

The search procedure followed Preferred Reporting Items for Systematic Reviews and Meta-Analyses (PRISMA) [51], and the flowchart was presented in Fig. 1. A total of 1339 articles were searched from three databases (392 articles from EBSCO, 594 articles from PubMed and 353 articles from Web of Science). A total of 878 articles remained after removing 461 duplicates by checking the title and/or abstract. Furthermore, based on the inclusion and exclusion criteria, two authors screened 25 full-text articles for the final selection. Finally, 16 studies [5, 6, 46, 47, 49, 50, 52–61] met the inclusion criteria for this review.

### Characteristics of studies and sample

**Fig. 2:**
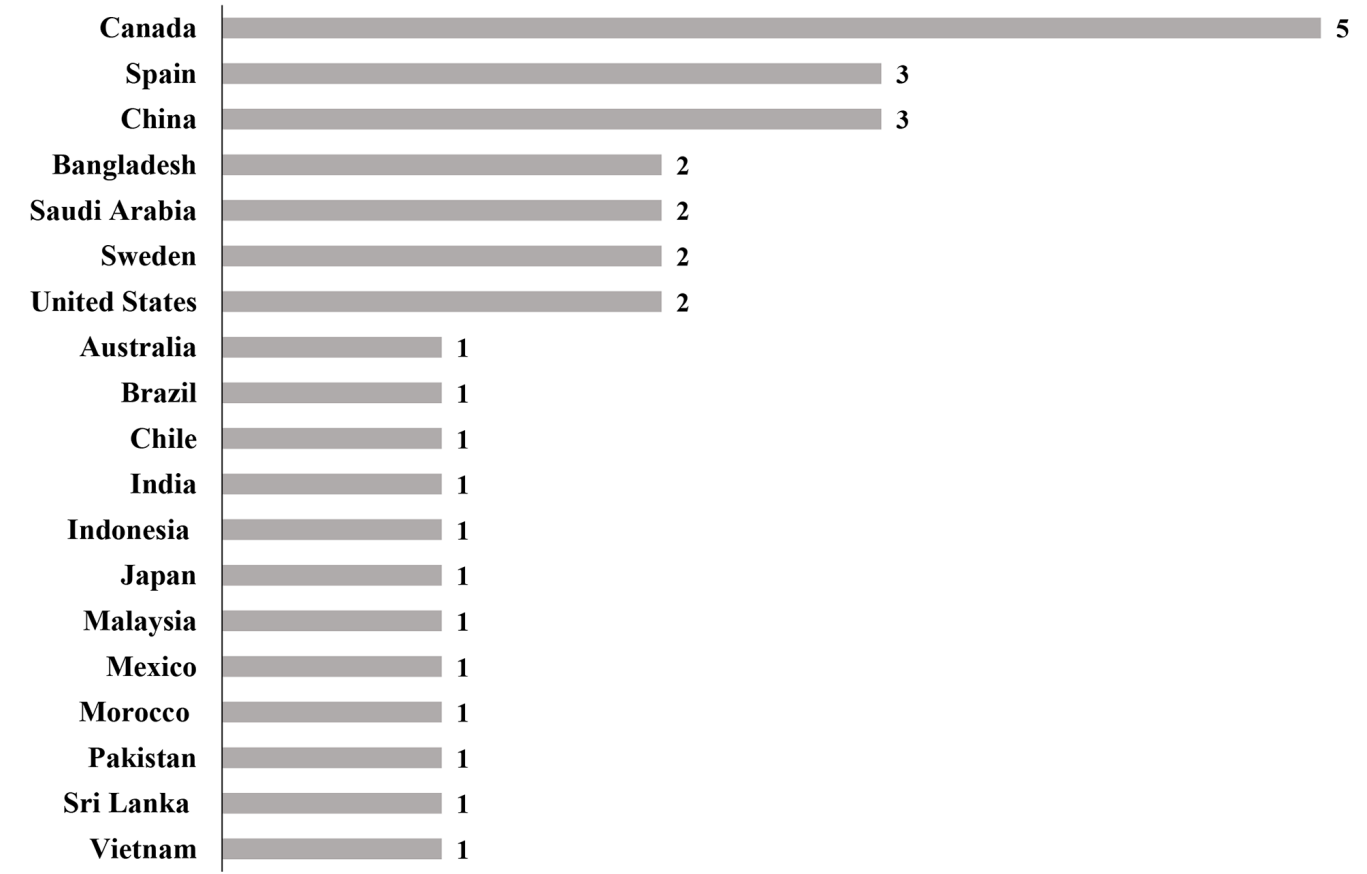
Number of publications involved in studies using 24-hour movement guidelines during the COVID-19 pandemic by country from the included studies of this review

Figure 2 illustrated populations from nineteen different countries extracted from fifteen included studies. Five studies focused on populations from Canada [5, 6, 47, 54, 55]. Three studies targeted populations from Spain [50, 59, 60] and China [58, 60, 61]. Two studies included populations from Bangladesh [57, 60], the United States [49, 60], Saudi Arabia [52, 53], Sweden [56, 60]. In addition, three studies included populations from different countries [49, 59, 60], of which populations from fourteen countries (Australia, India, Indonesia, Malaysia, Morocco, Pakistan, Sri Lanka, Vietnam, Japan, Chile, Mexico, Sweden, China and Brazil) were all extracted from one study [60], López-Gil’s study [59] included populations from Spain and Brazil, and Jáuregui’s study [49] included populations from Chile, Mexico, and the United States.

The characteristics of the studies included in this review were summarized in Table 1. In terms of study design, ten studies (62.5%) were cross-sectional designs [5, 6, 49, 52, 53, 55–59, 61], and four studies (25.0%) were repeated cross-sectional designs [47, 50, 52, 54]. Two studies (12.5%) were longitudinal in design [46, 60]. In regard to sample size, two studies (12.5%) had a sample size under 100 [54, 57], four studies (25.0%) had a sample size between 100 and 1000 [46, 50, 56, 60], and ten studies (62.5%) had a sample size above 1000 [5, 6, 47, 50, 52–55, 59, 61]. Regarding age groups, seven studies (43.8%) included preschool students (under 5 ys) [46, 49, 56, 57, 59–61]. Ten studies (62.5%) included children (aged 5–13 ys) [5, 6, 47, 50, 52–55, 59, 61], and seven studies (43.8%) included adolescents (aged 14–17 ys) [5, 6, 47, 50, 54, 55, 59]. Only one study (6.3%) targeted adults (18 + ys) [58]. Fifteen studies (93.8%) assessed the general population [5, 6, 46, 47, 49, 50, 52, 53, 55–61], and only one study (6.3%) assessed people with disability or disorder [54].

**Table 1:** Characteristics of studies included in this review

Categorization		N (%)	Studies
*Study design*			
	Repeated cross-sectional study	4 (25.0%)	Alanazi et al.,2022a, Angel et al.,2022, Arbour-Nicitopoulos et al.,2022, Moore et al.,2021
	Cross-sectional study	10 (62.5%)	Alanazi et al.,2022b, Caldwell et al.,2022, Delisle et al.,2020, Feng et al., 2022, Hossain et al.,2021, Jáuregui et al.,2022, Liang,2021, López-Gil et al.,2021, Moore et al.,2020, Mitra et al. 2020
	Longitudinal study	2 (12.5%)	Hyunshik et al.,2021, Okely et al.,2021
*Simple size*			
	< 100	2 (12.5%)	Arbour-Nicitopoulos et al.,2022, Hossain et al.,2021
	≥ 100, ≤ 1000	4 (25.0%)	Angel et al.,2022, Delisle et al.,2020, Hyunshik et al.,2021, Okely et al.,2021
	> 1000	10 (62.5%)	Alanazi et al.,2022a, Alanazi et al.,2022b, Caldwell et al.,2022, Feng et al., 2022, Jáuregui et al.,2022, Liang,2021, López-Gil et al.,2021, Mitra et al.2020, Moore et al.,2020, Moore et al.,2021
Age group*			
	Preschool (under 5 ys)	7 (43.8%)	Delisle et al.,2020, Feng et al., 2022, Hossain et al.,2021, Hyunshik et al.,2021, Jáuregui et al.,2022, López-Gil et al.,2021, Okely et al., 2021
	Children (aged 5–13 ys)	10 (62.5%)	Alanazi et al.,2022a, Alanazi et al.,2022b, Angel et al., 2022, Arbour-Nicitopoulos et al.,2022, Caldwell et al., 2022, Feng et al., 2022, López-Gil et al.,2021, Mitra et al.2020, Moore et al., 2020, Moore et al., 2021
	Adolescent (aged 14–17 ys)	7 (43.8%)	Angel et al.,2022, Arbour-Nicitopoulos et al.,2022, Caldwell et al.,2022, López-Gil et al.,2021, Mitra et al.2020, Moore et al.,2020, Moore et al.,2021
	Adult (18 + ys)	1 (6.3%)	Liang et al.,2021
*Healthy population or those with disability*			
	General people	15 (93.8%)	Alanazi et al.,2022a, Alanazi et al.,2022b, Angel et al.,2022, Caldwell et al.,2022, Delisle et al.,2020, Feng et al., 2022, Hossain et al.,2021, Hyunshik et al.,2021, Jáuregui et al.,2022, Liang,2021, López-Gil et al.,2021, Mitra et al.2020, Moore et al.,2020, Moore et al.,2021, Okely et al.,2021
	People with disability/disorder	1 (6.2%)	Arbour-Nicitopoulos et al.,2022

Note: *Studies included different age groups

### Measurement and Assessment of studies

**Fig. 3:**
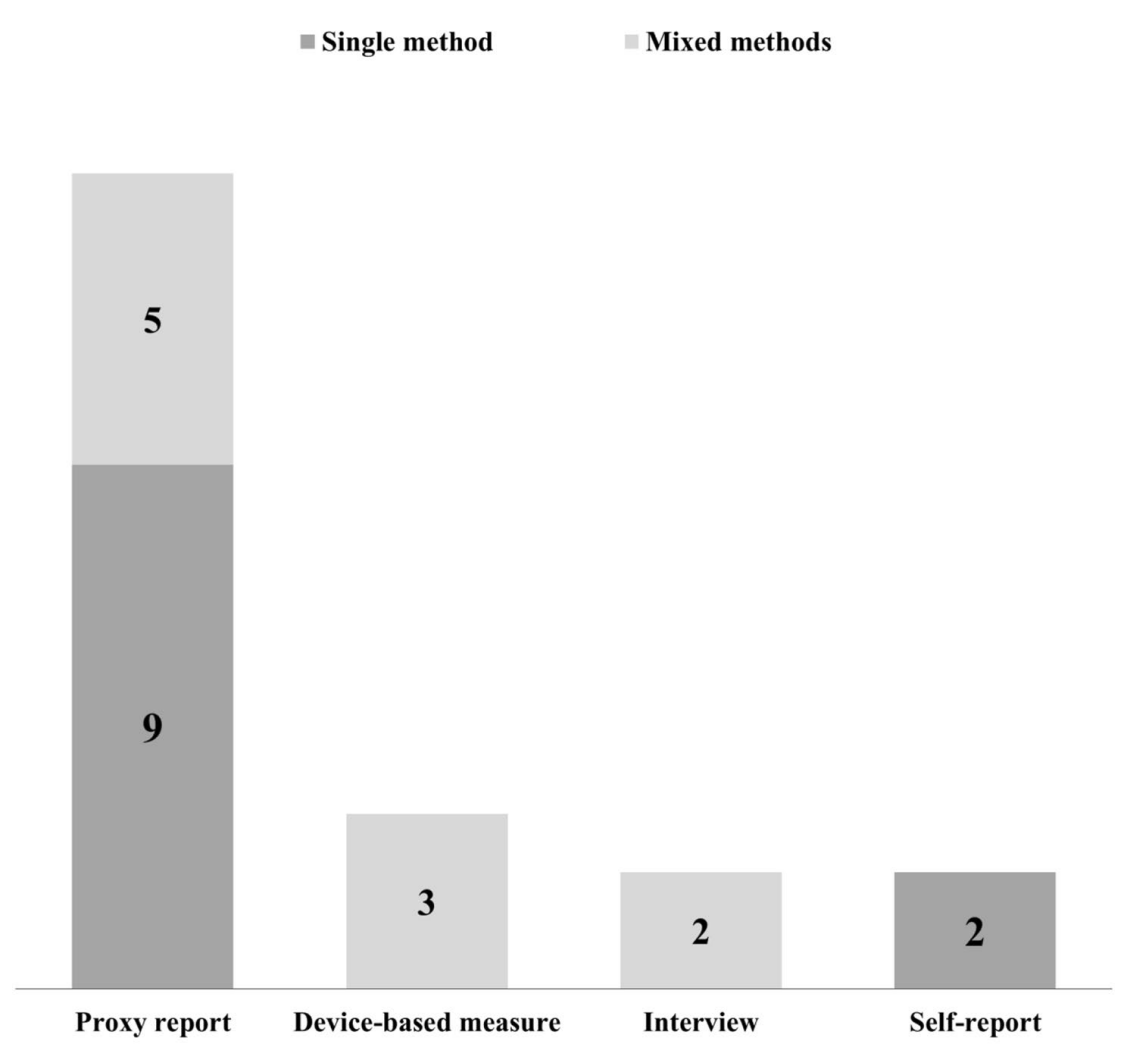
Number of studies according to the measurement and assessment used. Notes: single method refers to only use one measure tool in study survey; mixed methods refer to use ≥ 2 measure tools in study survey

The measurement and assessment methods used in the studies were provided in Fig. 3. Eleven studies used a single method [5, 6, 47, 49, 50, 52, 53, 55, 58, 59, 61], of which Nine studies applied a proxy report assessment [5, 6, 47, 49, 52, 53, 55, 59, 61] and two studies applied a self-report measurement [50, 58]. Additionally, ten studies used mixed methods, of which five studies applied a proxy report assessment [46, 54, 56, 57, 60], three studies [46, 56, 57] used a device-based measurement (accelerometer), and two studies [50, 58] applied an interview measurement.

### Categorization of studies using the 24-hour movement guidelines

Table 2 shows that the studies from the perspective of the VIRTUE framework. Three research areas (outcomes, correlates/determinants, and time-use composition) were considered in this review. In regard to outcomes, ten studies (62.5%) included sociodemographics (e.g., gender/sex, age, region, urban/rural, country income level) [5, 47, 49, 52, 53, 55–57, 59, 60]. Three study [6, 54, 60] included behaviours/lifestyle (e.g., outdoor activity) [6], disability [54], environment (e.g., presence of outdoor space within house compound) [60], and social and cultural factors (e.g., the parent’s concern about the child’s movement behaviour, receiving any support from their childcare center, the parent’s perceived ability to support the child in having healthy movement behaviours, the parent’s perceived level of stress, the parent’s perceived level of exhaustion) [60]. Regarding the research area of correlates/determinants, two studies focused on psychological factors (e.g., depression, anxiety and stress) [58, 61]. In the research area of time-use composition, all included studies assessed the prevalence of meeting the 24HMG. Eight studies (50.0%) included trends in meeting the 24HMG [46, 47, 49, 50, 52, 54, 59, 60], of which two studies [46, 50] included different numbers of participants meeting the 24HMG.

**Table 2:** Number of studies according to the Framework for Viable Integrative Research in Time-Use Epidemiology

Research areas	Domains	N (%)	Studies
Outcomes	Socio-demographics	10 (62.5%)	Alanazi et al., 2022a; Alanazi et al., 2022b; Caldwell et al., 2022; Hossain et al., 2022; Delisle Nyström et al., 2022; Jáuregui et al., 2022; López-Gil et al., 2021; Moore et al., 2020; Moore et al., 2021; Okely et al., 2021
	Behaviours/lifestyle	1 (6.3%)	Mitra et al., 2020
	Disability	1 (6.3%)	Arbour et al., 2022
	Environment	1 (6.3%)	Okely et al., 2021
	Social and cultural	1 (6.3%)	Okely et al., 2021
Correlates/Determinants	Psychological factors	2 (12.5%)	Liang et al., 2021, Feng et al., 2022
Time-use composition	Prevalence	16 (100%)	Hyunshik, et al., 2021, Angel et al., 2022, Alanazi et al., 2022a, Alanazi et al., 2022b, Caldwell et al., 2022, Feng et al., 2022, Hossain et al., 2022, Delisle Nyström et al., 2022; Arbour-Nicitopoulos et al., 2022, López-Gil et al., 2021, Okely et al., 2021, Moore et al., 2021, Alanazi et al., 2022a, Jáuregui et al., 2022, Mitra et al., 2020, Okely et al., 2021
	Trends	8 (50.0%)	*Hyunshik, et al., 2021, Angel et al., 2022*, Arbour-Nicitopoulos et al., 2022, López-Gil et al., 2021, Okely et al., 2021, Moore et al., 2021, Alanazi et al., 2022a, Jáuregui et al., 2022

Note: VIRTUE framework = The Framework for Viable Integrative Research in Time-Use Epidemiology; Italic refers to studies which met different number of guidelines

### Levels and chagnes in the prevalence of meeting the 24-hour movement guidelines

**Fig. 4:**
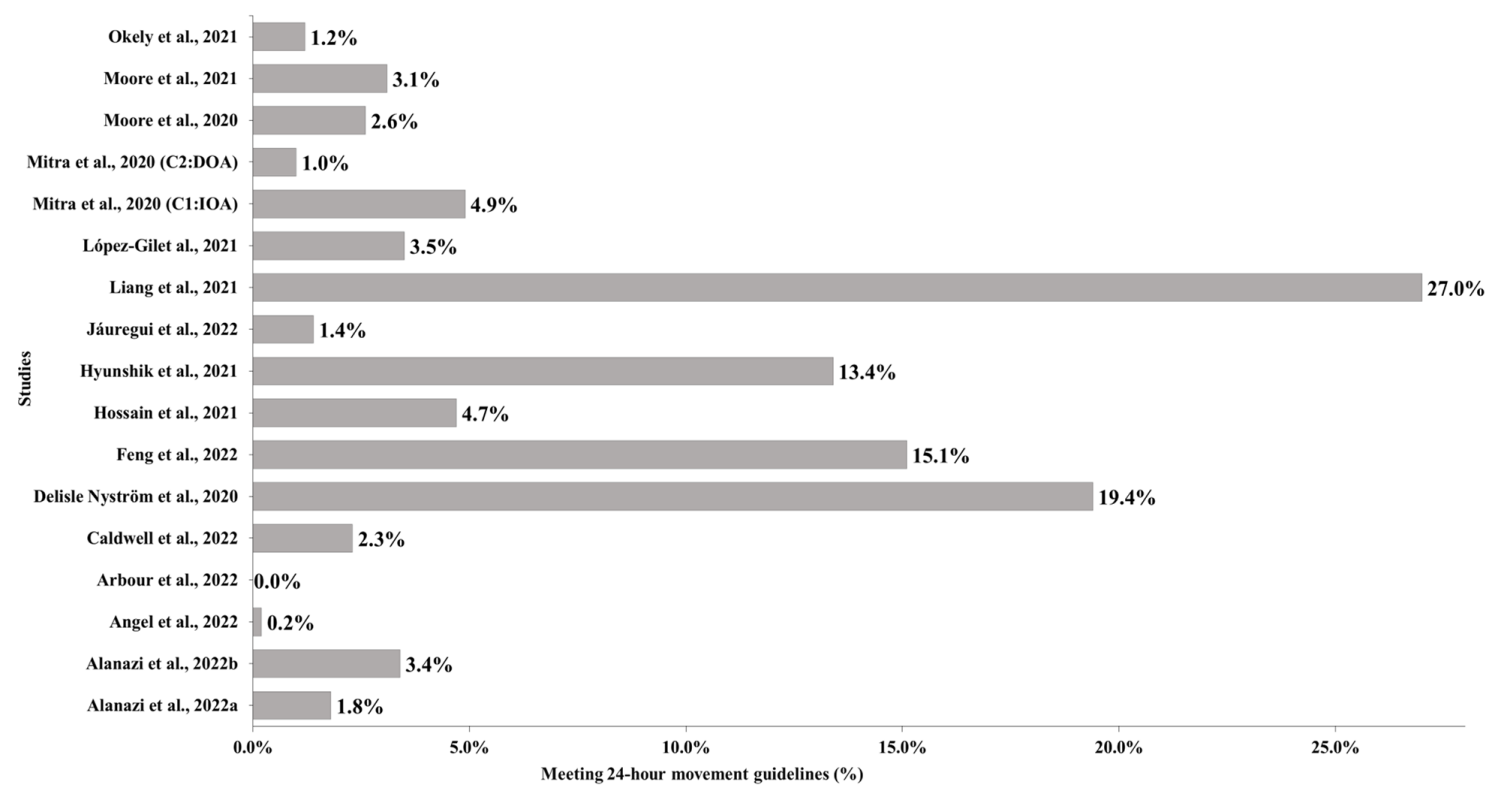
Prevalence of meeting 24-hour movement guidelines during the COVID-19 reported by studies. Note: CI: IOA = cluster 1: increase outdoor activity; C2: DOA = cluster 2: decrease outdoor activity

As shown in Fig. 4, seventeen outcomes were extracted from sixteen included studies (one study included two outcomes). Thirteen (76.5%) results (from twelve studies) [5, 6, 47, 49, 50, 52–55, 57, 59, 60] showed that the prevalence of meeting the 24HMG was under 5% during the COVID-19 pandemic, of which two studies reported a percentage less than 1% [50, 54]. Four studies reported that more than 10% of the population (13.4%, 15.1%, 19.4%, and 27.9%, respectively) met the 24HMG during the COVID-19 pandemic period [46, 56, 58, 61]. During the pandemic, the highest prevalence of meeting the 24HMG was 27.0% [58], and the lowest was 0.0% [54].

As shown in Table 3, eight outcomes from seven studies reported that the percentage of the population meeting the 24HMG was decreased [46, 47, 49, 50, 52, 59, 60], of which half of the studies [47, 49, 50, 52] reported a significant decrease from prior to and during the COVID-19 pandemic. Three studies showed a decrease of more than 3% [49, 50, 59]. One study reported a percentage of 0.0% with no change [54]. Nonsignificant increases were reported both in children and youth (only girls) [47].

**Table 3:** The changes in prevalence of meeting 24-hour movement guidelines reported by the included studies

Authors/year		Before	During	Code	Summary
Arbour-Nicitopoulos et al., 2022		0.0%^#^	0.0%	NC	/
López-Gil et al., 2021		6.9%	3.5%	D-	D-
Hyunshik et al., 2021		15.6%	13.4%	D-	
Okely et al., 2021		1.4%	1.2%	D-	
Moore et al., 2021	children-boys	6.5%^#^	4.3%	D-	
	children-girls	2.8%^#^	4.6%	I^+^	/
	youth-girls	0.8%^#^	1.3%	I^+^	
	youth-boys	0.5%^#^	2.4%	D-^*^	D-*
Alanazi et al., 2022a		3.4%^#^	1.8%	D-^*^	
Angel et al., 2022		3.3%	0.2%	D-^*^	
Jáuregui et al., 2022		4.8%	1.6%	D-^*^	

Note: ^#^Studies survey during COVID-19 as Time 1 reported; D-^*^ = significant decrease, D- = decrease but not report *p* value/non-significant decrease; I^+^ = non-significant increase; NC = no change

## Discussion

The aim of this review was to synthesize the evidence from studies using the 24HMG during the COVID-19 pandemic, presenting a knowledge map and research landscape. The main findings of this review were as follows: first, the number of studies using the 24HMG was very limited (n = 16), with most of the studies targeting children and adolescents as study population, and most of the studies used subjective measures and were cross-sectional (n = 14) and conducted in Western countries. Second, most studies found that during the COVID-19 pandemic, the prevalence of meeting the 24HMG was very low (11 studies reported below 4%), and it declined greatly compared with the prevalence before the COVID-19 pandemic. Third, according to the VIRTUE framework, studies focusing on the prevalence of meeting the 24HMG and the trends, as well as sociodemographic correlates of the prevalence, were dominant across the included studies. Evidence synthesized from this review can help inform future research development and policymakers to implement effective approaches against public health events.

### Overall status of studies and study characteristics

The findings of this review demonstrated a very limited number of studies using the 24HMG during the COVID-19 pandemic. Compared with studies investigating the prevalence of meeting the PA, SB, or sleep guidelines [30–35] in insolation, the number was largely lower, which in part reflects less research attention and interest in 24HMG studies. Some possible reasons can be proposed. The first one was that research within 24HMG has had a relatively short history compared with PA, SB and sleep studies in insolation, so the number of 24HMG studies cannot be as large as possible. Second, it might be difficult to collect data on PA, SB and sleep simultaneously during the COVID-19 pandemic. Despite the limited number, those included studies could also advance the knowledge around health behaviour research during the pandemic and provide evidence to refine the 24HMG in the future.

The majority of the studies included in this review were cross-sectional and conducted in Western countries. Similar findings on PA or SB studies in insolation were found [7], which is consistent with the current review. This was likely because a cross-sectional study may be the most economical and feasible study design during the COVID-19 pandemic period. During the isolation period, it was very challenging to conduct longitudinal and interventional studies on 24-hour movement behaviours owing to social distancing and lockdown. Additionally, some measures against the pandemic also had impacts on measures used by the studies included in this review. As observed in this review, most of the included studies used subjective measures to collect data on movement behaviours, which was likely due to social distancing and lockdown as well [8, 9]. However, with the increasing use of device-based measures of movement behaviours, such as mobile phones or some other individually used technological devices (e.g., smartphones, wearable activity monitors) [62, 63], future research should consider utilizing these devices to capture more accurate data on movement behaviours [7, 63].

Researchers from Western countries were the main contributors to 24HMG studies during the COVID-19 pandemic. Previous studies have shown that research in PA [15] and SB research [7], and even sleep research [34], mainly comes from Western countries [7, 34, 64], which can to some extent explain this finding in our review. Such a bias has been observed in other health-related fields [65]. Similarly, research bias was also found in terms of the study population. In the current review, all the included studies focused on children and adolescents. This was likely due to two reasons, of which the first was that children and adolescents were the prioritized studied population in PA-related research [66] and the second was that the first 24HMG was designed for children and adolescents [24]. However, the first 24HMG for adults was released in 2020 [24]. Collectively, the above reasons could explain why the included studies prioritized children and adolescents rather than adults or other study populations.

### Low levels of 24-hour movement behaviours

The results indicated a low prevalence of meeting the 24HMG (most of the included studies reported below 5%) in children and adolescents during the COVID-19 pandemic, and a significant decline in the prevalence was found compared with the pre-COVID-19 pandemic. This finding was expected on the basis of previous studies and evidence bases. Owing to social distancing and lockdown policies during the COVID-19 pandemic, individuals’ low levels of PA [14–16], high levels of SB [17–19] and worse sleep [50] jointly contributed to the low prevalence of meeting the 24HMG in children and adolescents. The implementation of restrictions has resulted in a rise in “stay-at-home” holidays. A previous study indicated that children’s sleep, SB, and PA exhibit lower levels of regulation on unstructured days (e.g., school holidays) in comparison to structured days (e.g., school days) [67]. Furthermore, this lack of regulation has been associated with a rise in the prevalence of children having electronic screen devices in their bedrooms, leading to inadequate sleep duration and PA [52].

Despite four studies reported that the prevalence of meeting the 24HMG over 10% [46, 56, 58, 61], overall low level was still exhibited. The potential reasons could be attributed to variations of survey population and study recruitment, with three studies specifically examining preschool children and one study concentrating on college students. In the case of preschool children, their limited awareness of the pandemic might influence the outcomes. Conversely, college students may have established similar daily routines, whether residing in dormitories or at home. Additionally, the study reported that the samples were recruited using a convenience sampling procedure [58], which may also influence the research findings.

### Change of 24-hour movement behaviours

Our findings mainly suggested a significant decline in the prevalence of meeting the 24HMG before and after the COVID-19 pandemic, even though one study reported the findings of increasing prevalence in some subgroups of children and adolescents [47]. This difference may be attributed to variations in virus transmission waves and the implementation of strict lockdown policies across different countries, as well as differences in the timing of measurements in studies [47, 50]. These findings indirectly illustrated the impact of pandemic restrictions on individuals’ 24-hour cycle behavior. There has been a perceived decline in young people’s behaviors following the COVID-19 pandemic, with significant changes observed in PA levels, recreational screen time, and sleep duration [50]. Given the measures against the COVID-19 pandemic, children and adolescents’ access to activity facilities and opportunities was reduced [69]. Moreover, owing to home quarantine, increased SB, including recreational screen time and domestic sitting time, has been observed [7]. These factors may also negatively impact on sleep [70]. Furthermore, the closure of schools during both strict and mild confinement reduces children and adolescents’ access to and opportunities for PA, such as physical education classes and organized PA [71, 72]. Additionally, the COVID-19 pandemic has also been associated with a decrease in outdoor playtime among children and adolescents [5, 73]. Spending less time outdoors has a substantial impact on PA and SB, further reducing the prevalence of meeting the 24HMG [5]. As the restrictions imposed due to the COVID-19 pandemic are gradually lifted, it is crucial to address and mitigate the negative impacts on 24-hour movement behaviours in youth. Future research should focus on understanding the long-term consequences of the pandemic on children and adolescents’ movement behaviors. This includes investigating the effectiveness of interventions aimed at promoting PA, reducing SB, and improving adherence to the 24HMG. Furthermore, it is imperative to investigate strategies that enhance access to recreational facilities, encourage outdoor play, and offer organized PA opportunities amidst persistent public health challenges. This will enable the development of evidence-based interventions and policies aimed at promoting the health and well-being of children and adolescents in the aftermath of the pandemic.

### Research topics of the 24HMG studies during the pandemic

On the basis of the VIRTUE framework formulated by Pedisic et al. [45], we examined the research topic of included studies conducted during the COVID-19 pandemic. The findings suggested that studies on time-use compositions and correlates were predominant. This situation can also be observed in PA, SB and sleep epidemiology research in insolation [13, 74], which was similar to our review. One possible explanation for this finding is that these two domains of study can be conducted with relatively low testing burden and are easier for researchers to design and perform. Given the constraints and limitations imposed by the pandemic, it is understandable that researchers gravitated towards areas where data collection and analysis could be carried out more easily. Among the time-use composition studies, numerous investigations have reported changes in the prevalence of meeting the 24HMG before and during the COVID-19 pandemic. However, it is equally important to direct research efforts towards examining the changes in post COVID-19 conditions. Such investigations would provide valuable insights into the impact of the pandemic on population health and inform strategies for the future. By expanding research beyond the immediate effects of the pandemic, we can gain a comprehensive understanding of the long-term implications on individuals’ time-use compositions and their adherence to the 24HMG. This knowledge will be crucial for developing targeted interventions and policies that promote healthier time-use behaviors in the post-pandemic era.

In terms of the correlates examined in the 24HMG studies conducted during the COVID-19 pandemic, the majority focused on sociodemographic factors, while a few studies assessed factors of other domains, such as social, cultural, and environmental factors. This finding is consistent with previous studies [34], partly because of relatively easy data collection on sociodemographic factors. Furthermore, this finding was also similar to the evidence from 24HMG research conducted before or after the COVID-19 pandemic [7]. This suggests that the emphasis on sociodemographic factors in 24HMG studies is not solely influenced by the pandemic but has been a prevailing trend in the field. Based on our review and the existing literature, it is evident that future studies should aim to explore a broader range of factors influencing 24HMG from different domains. By expanding the scope of investigation beyond sociodemographic factors, researchers can gain a more comprehensive understanding of the complex interplay between activity behavior and various contextual factors. This will contribute to a more nuanced understanding of the relationship between 24HMG and its determinants, ultimately informing interventions and strategies to improve individuals’ health and well-being.

Two study treated 24-hour movement behaviours as correlated and assessed their association with mental health outcomes among Chinese populations of preschool and university students [58, 61]. In contrast, the number of studies conducted before or after the COVID-19 pandemic largely exceeded the number. Despite the limited number, evidence can also be used for future refinement and update of the 24HMG for children and adolescents. Based on prior evidence has demonstrated that the low prevalence of meeting the 24HMG was in part responsible for undesirable health outcomes in children and adolescents, such as psychological outcomes (e.g., depression and anxiety) [58] and physical outcomes (e.g., cardiometabolic risk and adiposity) [68]. Future research can adopt longitudinal designs to examine the long-term effects of 24-hour movement behaviours on mental health in different age group (preschool students, children and adolescents, and adults) By tracking individuals’ behaviors and mental health outcomes (at different stages before, during and after pandemic), researchers can gain insights into the potential causal relationships and identify whether these associations persist over time. This research will contribute to providing valuable recommendations for the future development of human behavior and psychology.

### Strength and limitations

This study’s strengths included a comprehensive review of the prevalence of 24HMG during the pandemic and its analysis of the changes in 24HMG before and after the outbreak. Additionally, it provides a summary of the research topics based on the VIRTUE framework. However, there were some limitations that should be acknowledged. Firstly, most of the studies were cross-sectional studies, which had an impact on the change of meeting 24HMG during the COVID-19 pandemic. Secondly, the included English studies of this review were only searched in three common databases, written in other languages articles were not included. Thirdly, this study did not incorporate COVID-19 policies, however, it is beneficial to analyse the impact of policies on meeting 24HMG in the future study. Additionally, this study did not classify and review adherence to 24HMG among genders, countries with varying socioeconomic status, and different age groups. Future targeted reviews (e.g., focusing on children and adolescents) are also valuable as these would facilitate interventions or policy development. Finally, this study just summarized the research topics of 24-hour movement behaviours during the COVID-19 based on the VIRTUE framework. Future studies are recommended to explore (through systematic review, meta-analysis., etc.) the results of relationships between different factors and 24-hour movement behaviors based on VIRTUE framework.

## Conclusion

This review summarized the evidence from studies using 24HMG during the COVID-19 pandemic, offering a knowledge base for future research and policy development. Based on the findings, the COVID-19 may tend to have a negative impact on the prevalence of meeting 24HMG among different age-group populations. According to the study characteristics and research domains, studies using the 24HMG have a large space for improvement in terms of study design, measurement protocols and study domains (e.g., correlates and health outcomes).

### Electronic supplementary material

Below is the link to the electronic supplementary material.

Supplementary Material 1

## Data Availability

The datasets used and/or analysed during the current study available from the corresponding author on reasonable request.

## Abbreviations

24HMG: 24-Hour Movement Guidelines
VIRTUE: Viable Integrative Research in Time-use Research
PA: Physical activity
SB: Sedentary behaviour
WHO: World Health Organization
