# Supplementary material for: 24-Hour movement behaviours research during the COVID-19 pandemic: a systematic scoping review
Source: BMC Public Health. 2023 Nov 7;23:2188. doi: 10.1186/s12889-023-17136-y (PMC10631189; doi:10.1186/s12889-023-17136-y)
Supplement: Supplementary file 1 — Supplementary Material 1 [file 12889_2023_17136_MOESM1_ESM.docx]

**Supplementary material of search strategy**

**Search words in three categories:**

| *(1)* | *(2)* | *(3)* |
| --- | --- | --- |
| *24-h** | *Guideline** | *COVID-19”* |
| *24 hour* | *recommendation** | *Coronavirus Disease* |
| *24-hour* |  | *Coronavirus* |
| *Movement Behavio** |  | *SARS-CoV-2* |
| *Sleep** |  | *nCoV* |
| *Screen”* |  |  |
| *Physical Activity* |  |  |

**Combine set with Booleans:**

(24-h* OR 24 hour OR 24-hour OR Movement Behavio* OR Sleep* OR Screen OR Physical Activity) AND (Guideline* OR recommendation*) AND (COVID-19 OR Coronavirus Disease OR Coronavirus OR SARS-CoV-2 OR nCoV)

**Refine option:**

Publication Date: 1 January 2020 to 30 November 2022

Document Types: article

Languages: English

***Due to the differences in databases,*** ***field tags of “Title”, “Abstract” and “Title/Abstract” were used in document retrieval.***

**Web of science:**

*(24-h* OR 24 hour OR 24-hour OR Movement Behavio* OR Sleep* OR Screen OR Physical Activity) (Title) and (Guideline* OR recommendation*) (Abstract) and (COVID-19 OR Coronavirus Disease OR Coronavirus OR SARS-CoV-2 OR nCoV) (Abstract) and English (Languages) and Article (Document Types) and 2020 or 2021 or 2022 (Publication Years)*

**EBSCO:**

*TI ( 24-h* OR 24 hour OR 24-hour OR Movement Behavio* OR Sleep* OR Screen OR Physical Activity ) AND AB ( Guideline* OR recommendation* ) AND AB ( COVID-19 OR Coronavirus Disease OR Coronavirus OR SARS-CoV-2 OR nCoV ) AND Publication Date (20200101-20221130) AND Publication Types (Academic Journal) AND Document Types (Article) AND Languages (English)*

**PubMed:**

*((24-h*[Title/Abstract] OR 24 hour[Title/Abstract] OR 24-hour[Title/Abstract] OR Movement Behavio*[Title/Abstract] OR Sleep*[Title/Abstract] OR Screen[Title/Abstract] OR Physical Activity[Title/Abstract]) AND (Guideline*[Title/Abstract] OR recommendation*[Title/Abstract])) AND (COVID-19[Title/Abstract] OR Coronavirus Disease[Title/Abstract] OR Coronavirus[Title/Abstract] OR SARS-CoV-2[Title/Abstract] OR nCoV[Title/Abstract]) Filters: Humans, English, from 2020/1/1 - 2022/11/30*
